# An inflammatory bowel disease-associated SNP increases local thyroglobulin expression to develop inflammation in miniature dachshunds

**DOI:** 10.3389/fvets.2023.1192888

**Published:** 2023-07-14

**Authors:** Yong Bin Teoh, Jing-Jing Jiang, Takeshi Yamasaki, Noriyuki Nagata, Toshiki Sugawara, Rie Hasebe, Hiroshi Ohta, Noboru Sasaki, Nozomu Yokoyama, Kensuke Nakamura, Yumiko Kagawa, Mitsuyoshi Takiguchi, Masaaki Murakami

**Affiliations:** ^1^Division of Molecular Psychoneuroimmunology, Institute for Genetic Medicine, Graduate School of Medicine, Hokkaido University, Sapporo, Japan; ^2^Laboratory of Veterinary Internal Medicine, Department of Clinical Sciences, Graduate School of Veterinary Medicine, Hokkaido University, Sapporo, Japan; ^3^Division of Molecular Neuroimmunology, Department of Homeostatic Regulation, National Institute for Physiological Sciences, National Institutes of Natural Sciences, Okazaki, Japan; ^4^Veterinary Teaching Hospital, Graduate School of Veterinary Medicine, Hokkaido University, Sapporo, Japan; ^5^Laboratory of Veterinary Internal Medicine, Department of Small Animal Clinical Sciences, School of Veterinary Medicine, Rakuno Gakuen University, Ebetsu, Japan; ^6^North Lab, Sapporo, Japan; ^7^Group of Quantum Immunology, Institute for Quantum Life Science, National Institute for Quantum and Radiological Science and Technology (QST), Chiba, Japan; ^8^Institute for Vaccine Research and Development (HU-IVReD), Hokkaido University, Sapporo, Japan

**Keywords:** miniature dachshund, canine, inflammatory colorectal polyps, SNP, thyroglobulin, inflammation, IL-6 amplifier

## Abstract

Inflammatory colorectal polyp (ICRP) in miniature dachshunds (MDs) is a chronic inflammatory bowel disease (IBD) characterized by granulomatous inflammation that consists of neutrophil infiltration and goblet cell hyperplasia in the colon. Recently, we identified five MD-associated single-nucleotide polymorphisms (SNPs), namely *PLG, TCOF1, TG, COL9A2*, and *COL4A4*, by whole-exome sequencing. Here, we investigated whether *TG* c.4567C>T (p.R1523W) is associated with the ICRP pathology. We found that the frequency of the T/T SNP risk allele was significantly increased in MDs with ICRP. *In vitro* experiments showed that TG expression in non-immune cells was increased by inducing the IL-6 amplifier with IL-6 and TNF-α. On the other hand, a deficiency of *TG* suppressed the IL-6 amplifier. Moreover, recombinant TG treatment enhanced the activation of the IL-6 amplifier, suggesting that TG is both a positive regulator and a target of the IL-6 amplifier. We also found that *TG* expression together with two NF-κB targets, *IL6* and *CCL2*, was increased in colon samples isolated from MDs with the T/T risk allele compared to those with the C/C non-risk allele, but serum TG was not increased. Cumulatively, these results suggest that the T/T SNP is an expression quantitative trait locus (eQTL) of *TG* mRNA in the colon, and local *TG* expression triggered by this SNP increases the risk of ICRP in MDs *via* the IL-6 amplifier. Therefore, *TG* c.4567C>T is a diagnostic target for ICRP in MDs, and *TG*-mediated IL-6 amplifier activation in the colon is a possible therapeutic target for ICRP.

## 1. Introduction

Inflammatory colorectal polyp (ICRP) in miniature dachshunds (MDs) is a chronic inflammatory disease confined to the colorectal region of dogs. It is characterized by multiple polypoid lesions or a single pedunculated granulomatous lesion growing from the colorectal mucosa, which has been reported only in Japan (1). Histopathologically, ICRP has been differentiated from other types of polyps by its traits of granulomatous inflammation with neovascularization, the infiltration of neutrophils, lymphocytes, and/or macrophages, goblet cell hyperplasia, exaggerated mucus production, and crypt dilation. Clinical signs of hematochezia, tenesmus, and mucoid feces have also been reported (1–3). ICRP responds well to immunosuppressive treatments such as leflunomide, prednisolone, and cyclosporine (4, 5). However, recurrences are common despite these treatments (2, 3), and further study of the ICRP pathogenesis is needed to develop a better cure. The inflammatory mechanism of ICRP has been reported to include the dysregulation of innate immunity, the upregulation of toll-like receptors (TLRs), and the overexpression of pro-inflammatory cytokines (6–10). Notably, MDs have a much higher tendency to develop ICRP, with an odds ratio of 24.63, compared to other dog breeds (1), signifying the importance of the genetic basis of this disease. The single-nucleotide polymorphisms (SNPs) of nucleotide-binding oligomerization domain 2 (*NOD2*) gene were investigated in the pathogenesis of ICRP but no major effect was found (11), leaving the detailed molecular mechanism of the inflammation unknown. Using whole-exome sequencing (WES), we found several genes related to inflammatory responses are MD-associated SNPs in ICRP (12).

Pioneer studies in chronic inflammation have shown that pro-inflammatory cytokine stimulation including IL-6 can enhance the expressions of inflammatory mediators in non-immune cells in the development of inflammatory diseases (13, 14). The simultaneous activation of NF-κB and STAT3 in non-immune cells has been shown to be the key molecular mechanism that enhances the activation of NF-κB. Although IL-6 is one of very few stimulators of STAT3, there are multiple stimulators of NF-κB, including TNF-α, IL-17A, growth factors, noradrenaline, and TLR ligands, during inflammation development (13–25). The combined effects of IL-6 and NF-κB on NF-κB activation were named the “IL-6 amplifier” (13). The IL-6 amplifier's role in the pathogenesis of inflammatory diseases has been well described in both mouse disease models and clinical samples of human patients (15, 17, 19, 26–38).

A genome-wide screening of the IL-6 amplifier identified approximately 500 target genes and 1,200 positive regulators (39). Recently, we used WES and found that some of these positive regulators, *PLG, TCOF1, TG, COL9A2*, and *COL4A4*, are MD-associated SNPs in ICRP (12). In this study, we verified that *TG* (thyroglobulin) is associated with the pathogenesis of ICRP. We found that MDs have a higher frequency of the *TG* risk allele T/T, which in turn increases *TG* expression in the colon, resulting in the activation of the IL-6 amplifier and chronic local inflammation. Furthermore, we found that TG deficiency suppressed and recombinant TG treatment enhanced the IL-6 amplifier activation in non-immune cells *in vitro*. Along with *TG*, the expression of other *NF-*κ*B* targets, such as *IL6* and *CCL2*, was concurrently increased in the colons of MDs with the risk allele T/T, strengthening the conclusion that TG is a potential therapeutic target for ICRP. These data uncover a new function of TG related to chronic inflammation and the breed specificity of ICRP in MDs.

## 2. Materials and methods

### 2.1. Animals

The genotyping of selected SNPs (Table 1) was performed by Sanger sequencing on 155 MDs diagnosed with ICRP, 90 age-controlled MDs without ICRP, 36 juvenile MDs aged < 1 year, and 40 dogs of other breeds. The details of the animals recruited and the samples used in this study are summarized in Supplementary Table 1. A DNA Blood & Tissue Kit (QIAGEN, Hilden, Germany) was used to derive genomic DNA (gDNA) from EDTA-archived blood, fresh colon tissue samples, or formalin-fixed paraffin-embedded colon tissue samples of the recruited dogs. Blood was collected by a veterinarian or a veterinary student supervised by a licensed veterinarian through a jugular venipuncture as part of routine blood workup for screening or diagnosis purposes. A signed consent form was obtained from the owners of all dogs for the tissue sample collection and use in this study.

**Table 1:** Chromosomal position and primers used for targeted genotyping.

**Gene**	**Chromosome number**	**Position**	**Genbank accession number**	**Variant (mRNA)**	**Variant (protein)**	**Forward primer (5^′^-3^′^)**	**Reverse primer (5^′^-3^′^)**
*TG*	13	29406010	NM_001048104	c.4567C>T	p.R1523W	GATGGGCGGTGAAGGGTTAA	GCACACAGCCCACAAGAAAG
*TG*-FFPE	13	29406010	NM_001048104	c.4567C>T	p.R1523W	ACCGACTGTCAGAGGAGTGA	TGACACTGGGAGTCGGTGA

### 2.2. Tissue sample collection

Endoscopic examinations were performed under general anesthesia for all dogs to retrieve the colon tissues. Each dog was administrated with midazolam (0.1 mg/kg) and butorphanol tartrate (0.2 mg/kg) intravenously as pre-medication and then with propofol (4 to 6 mg/kg) using the same route. Anesthesia was then maintained through the inhalation of isoflurane with oxygen, where additional butorphanol was administrated when necessary. Pulse oximetry readings, electrocardiograph, capnograph, arterial blood pressure, and rectal temperature were monitored throughout the anesthesia to ensure a smooth endoscopic procedure. Endoscopic procedures were completed within 2 h in all dogs, and all dogs recovered uneventfully. In MDs with ICRP, samples were collected as part of the diagnostic procedure, where polyp lesions deemed inflammatory on gross appearance were collected endoscopically with forceps or through electrosurgical snare polypectomy. Normal colorectal mucosae were collected endoscopically from the colorectal region adjacent to the diseased area based on gross appearance. At least six tissue samples were collected from both the polyp lesion site and normal colorectal mucosal region in MDs with ICRP. All biopsy specimens were assessed by a board-certified veterinary pathologist (YK) according to histopathological standards established by the World Small Animal Veterinary Association Gastrointestinal Standardization Group (40). The severity of ICRP inflammation in each case was staged according to a previous report (3). The information on the medication and histopathology for each group is shown in Supplementary Table 2. Each tissue sample was stored at −80°C for protein analysis or RNA*Later* RNA Stabilization Solution (Ambion Inc., Austin, TX, USA) for 24 h at 4°C to allow penetration and stabilization and then at −80°C for prolonged storage. Five specimens were collected for the qPCR analysis to perform a definitive diagnosis of ICRP.

### 2.3. Targeted genotyping

Polymerase chain reactions (PCRs) and Sanger sequencing were utilized to validate the genotype variants selected from WES data (12). The WES data have been deposited with links to BioProject accession number PRJDB16014 in the DDBJ BioProject database. Primer pairs used for the variant validation were designed using Primer3Plus software (http://www.bioinformatics.nl/cgi-bin/primer3plus/primer3plus.cgi; Table 1). Amplicons post-PCR were purified using a commercial clean-up reagent, ExoSAP-IT Express (Thermo Fisher Scientific, Waltham, MA, USA), and sequencing was performed using the Sanger method utilizing BigDye Terminator v3.1 (Thermo Fisher Scientific) and ABI PRISM 3100 Genetic Analyzer (Applied Biosystems, Waltham, MA, USA). FinchTV (https://digitalworldbiology.com/FinchTV) was used to interrogate the reads generated, and the allele frequency was compared between groups using Fisher's exact test.

### 2.4. Cell lines and stimulation conditions

The H4 human cancer cell line was purchased from ATCC (Sumitomo Dainippon Pharma, Osaka, Japan). All cell lines were cultured in DMEM (Thermo Fisher Scientific, Waltham, MA) enriched with 10% fetal bovine serum (Thermo Fisher Scientific) and treated with 1% penicillin and streptomycin at 37°C under 5% CO_2_. For the cytokine stimulation, cells were seeded into 96-well plates (1 × 10^4^ cells/well) and stimulated with human IL-6 (30 ng/ml; Toray Industries, Tokyo, Japan) plus human soluble IL-6 receptor (30 ng/ml; R&D Systems, Minneapolis, MN) and/or TNF- α (10 ng/ml; PeproTech, Tokyo, Japan) for 3 h after 2 h of serum starvation in Opti-MEM (Thermo Fisher Scientific, Waltham, MA). For the TG stimulation, the cytokine stimulation was modified to include cells stimulated with a 5x dilution of the cytokine with the serial addition of recombinant human TG (1 μg/ml, 5 μg/ml, and 10 μg/mL) as per the manufacturer's recommendation (Cloud-Clone Corp., USA). After stimulation, the cells were lysed, and the total RNA was retrieved for real-time PCR.

### 2.5. Quantitative real-time PCR in H4 cells

The Bio-Rad CFX96 real-time PCR system (Bio-Rad Laboratories, Hercules, CA, USA) and THUNDERBIRD SYBR quantitative PCR (qPCR) Mix (TOYOBO Co. Ltd., Osaka, Japan) were used to quantify the levels of target mRNA and internal control mRNA (glyceraldehyde-3-phosphate dehydrogenase, *GAPDH*). Total RNA was prepared from cells using a SuperPrep Cell Lysis Kit for qPCR (TOYOBO). The conditions for real-time qPCR were 40 cycles at 94°C for 15 s followed by 40 cycles at 60°C for 60 s. Relative *IL6* mRNA expressions were normalized to the level of *GAPDH* mRNA expression. Primers and their sequences for the qPCR are described in Table 2. For the *in vitro* experiments, all the experiments were performed in triplicate but also performed twice to ensure replication.

**Table 2:** Primer sequences used for qPCR in H4 cells.

**Gene**	**Forward primer (5^′^-3^′^)**	**Reverse primer (5^′^-3^′^)**
Human *IL6*	GGTACATCCTCGACGGCATCT	GTGCCTCTTTGCTGCTTTCAC
Human *GAPDH*	GAGTCAACGGATTTGGTCGT	CGCTCCTGGAAGATGGTG
Human *TG*	CCAGTGGCTTCTCTTCCTGACT	CCTTGGAGGAAGCGGATGGTTT

### 2.6. Human small-interfering RNAs

Human small-interfering RNAs (siRNA) were transfected into H4 cells using Lipofectamine RNAiMAX (Thermo Fisher Scientific). The sequences for the sense oligonucleotides of the knockdown constructs were human si-TG (1: CCUUAUGAGUUCUCACGGAtt and 2: GCUGCUACAUGGUAUUACUtt; Ambion Silencer Select siRNA, Thermo Fisher Scientific), human si-p65 (Ambion Silencer Select RELA siRNA, Thermo Fisher Scientific), and human siRNA negative control (Ambion Negative Control #1 siRNA, Thermo Fisher Scientific).

### 2.7. Enzyme-linked immunosorbent assay (ELISA) detection of serum *TG* levels

Serum samples from 22 MD-ICRP, 22 MD-Control, and 36 juvenile MDs were enrolled in this study. For the 44 adult MDs, blood samples were collected from April 2017 to April 2021 by a licensed veterinarian or a supervised veterinary student by venipuncture during the dogs' first visits to the Hokkaido University Veterinary Teaching Hospital (HUVTH) for routine diagnostics with written informed consent obtained from the owners. For the juvenile MDs, blood samples were collected by a licensed veterinarian (YBT) using a venipuncture. The juvenile MDs were owned by a small and medium enterprise pet store in Hokkaido, Japan; written informed consent was obtained from the enterprise's owner and veterinarian on duty, and sample collection was performed from November 2021 to February 2022. Serum samples separated after centrifugation were stored at −80°C until the TG analysis. Serum TG concentrations were measured as per protocol provided by the manufacturer using the Canine Thyroglobulin ELISA Kit (MyBioSource, San Diego, CA, USA, Catalog No: MBS2608140). The intra-assay coefficient of variability between sample replicates was < 20%. The information on the regional/systemic inflammation and medication (e.g., corticosteroids) is given in Supplementary Table 3.

### 2.8. Real-time quantitative PCR (qPCR) in canine colonic mucosa samples

For qPCR using canine colonic mucosa samples, seven MD-ICRP (three with T/T allele and four with C/C allele), which visited HUVTH between April 2017 and April 2021, were included; total RNA was extracted using an RNeasy Mini Kit (Qiagen, Valencia, CA, USA); and genomic DNA was removed using an RNase-free DNase Set (Qiagen) following the manufacturer's instructions. cDNA synthesis was then performed using oligo (dT) and M-MLV reverse transcriptase (Promega, Madison, Wisconsin, USA) from 1 μg total RNA as per the manufacturer's recommendation. Real-time TaqMan qPCR was performed using a commercially available set of a pre-designed probe and primers (Applied Biosystems) for *TG* (product no: Cf02701382_m1) with an endogenous control for colonic samples using *SDHA* (product no: Cf02664981_m1) confirmed by a previous study (41). Real-time qPCR was performed using TaqMan PCR probe master mix (KAPA Biosystems) and at the following cycle conditions: 40 cycles at 94°C for 3 s and 40 cycles at 60°C for 30 s. The relative *TG* mRNA expression levels were normalized to the level of *SDHA* mRNA expression. THUNDERBIRD SYBR qPCR Mix (TOYOBO Co. Ltd., Osaka, Japan) was used to quantify the levels of the target mRNA (*IL6, CCL2*) and internal control mRNA (succinate dehydrogenase complex, subunit A; *SDHA*). The condition for real-time qPCR was 40 cycles at 94°C for 15 s followed by 40 cycles at 60°C for 60 s. Relative *IL6* and *CCL2* mRNA expressions were normalized to the level of *SDHA* mRNA expression. The primers and their sequences for the qPCR are described in Table 3 and reported previously (42, 43). For the qPCR using these samples, all the experiments were performed in duplicates and performed twice to ensure replication.

**Table 3:** Primer sequences used for qPCR in canine colonic mucosa.

**Gene**	**Forward primer (5^′^-3^′^)**	**Reverse primer (5^′^-3^′^)**
*IL6*	TTAAGTACATCCTCGGCAAAATCT	CAGTGCCTCTTTGCTGTCTTCA
*SDHA*	GCCTTGGATCTCTTGATGGA	TTCTTGGCTCTTATGCGATG
*CCL2*	GAGTCACCAGCAGCAAGTGT	TGGGTTTGGCTTTTCTTGTC

### 2.9. Statistical analysis

Two-tailed Student's *t*-test and Fisher's exact test were used to analyze differences between the two groups. Values of *P* < 0.05 were considered significant.

## 3. Results

### 3.1. *TG* c.4567c>t (p.1523r>w) allele is increased in MDs with ICRP

**Figure 1:**
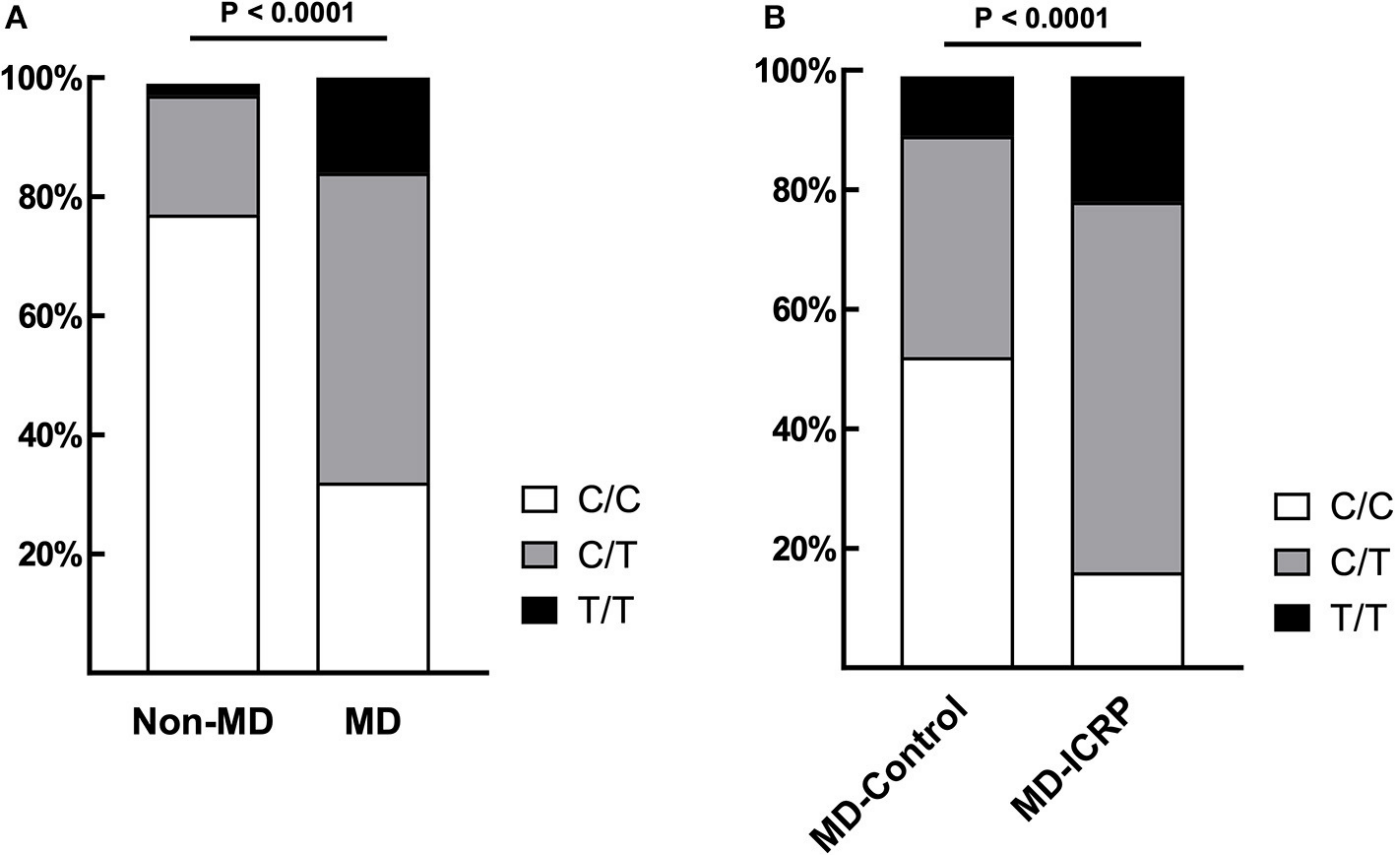
*TG* SNP is associated with ICRP in MDs. **(A)** The frequency of variant *TG* c.4567C>T (p.R1523W) was significantly higher in MDs (*n* = 281) when compared to other breeds (*n* = 40). *p* < 0.0001. **(B)** Among MDs, age-matched, case–controls showed that the same variant was significantly higher in MD-ICRP (*n* = 155) than MD-Control (*n* = 90). *p* < 0.0001.

The frequency of *TG* c.4567C>T (p.1523R>W) allele in MDs (*n* = 281) was 69.04% (48 homozygous and 246 heterozygous), which was significantly higher than in dogs of other breeds (22.5%; one homozygous and eight heterozygous, *n* = 40) (*P* < 0.0001, Figure 1A). Sequence chromatograms of the target variant are shown in Supplementary Figure 1A. Furthermore, the frequency of the T risk allele (84.52%) was significantly higher in MD-ICRP (median age = 10 years old; 34 homozygous, 97 heterozygous, *n* = 155) than in MD-controls (47.78%; median age = 12 years old; 9 homozygous, 34 heterozygous, *n* = 90) (*P* < 0.0001, Figure 1B). Thus, the *TG* c.4567C>T (p.1523R>W) allele was increased in MDs with ICRP. Finally, we found that one amino acid of the *TG* gene was conserved in dogs and other mammals (Supplementary Figures 1B, C).

### 3.2. *TG* is a positive regulator and target of the IL-6 amplifier activation

**Figure 2:**
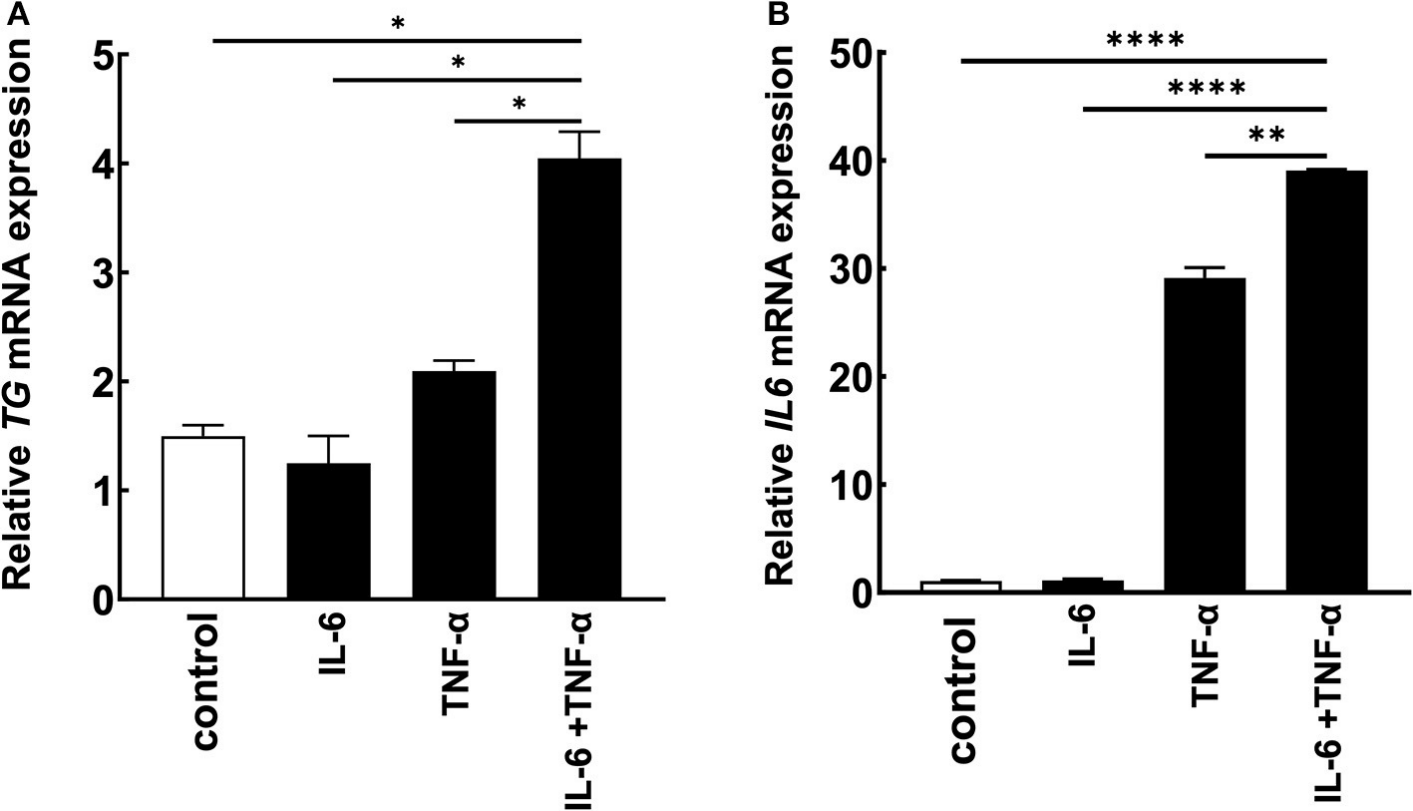
*TG* is a potential target of the IL-6 amplifier *in vitro*. **(A, B)** H4 cells were stimulated with cytokines, and *IL6* and *TG* levels were assessed. Means ± standard deviations are shown. *p < 0.05, **p < 0.01, ****p < 0.0001.

**Figure 3:**
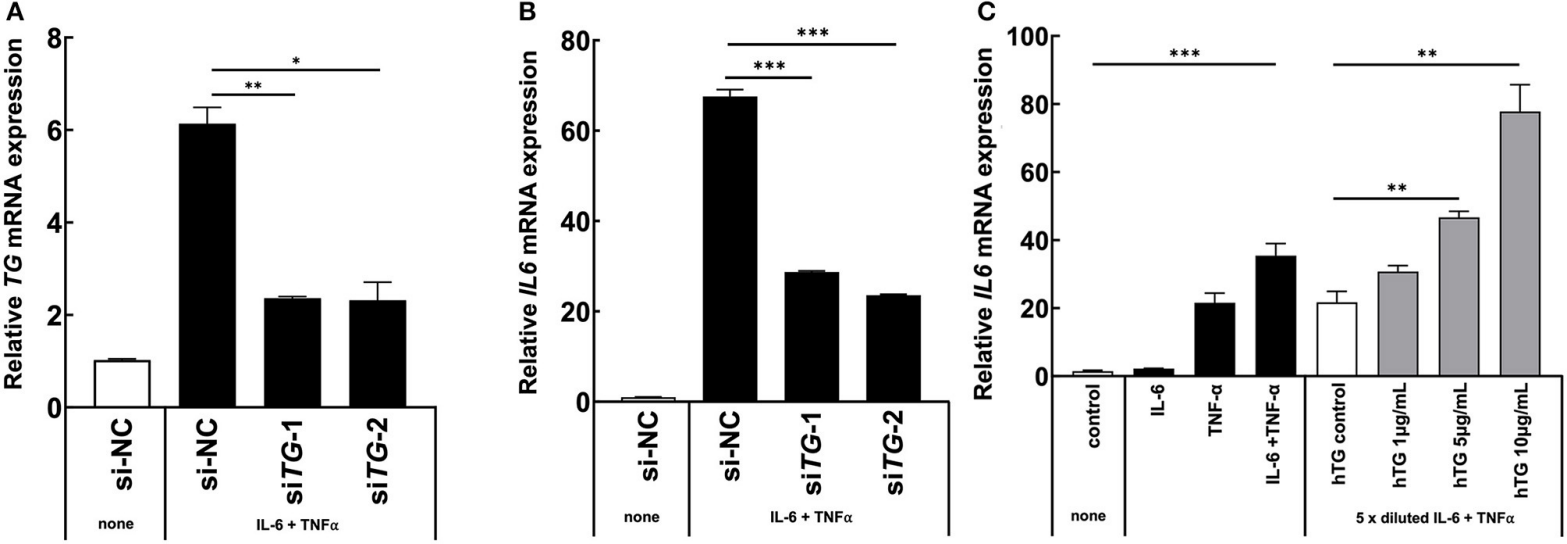
TG is critical for IL-6 amplifier activation *in vitro*. **(A, B)** H4 cells were transfected with two different siRNAs for all *TG* variants or a siRNA negative control. *IL6* levels and *TG* knockdown efficiency were assessed. Means ± standard deviations are shown. *p < 0.05, **p < 0.01, ***p < 0.005. **(C)** H4 cells were stimulated with cytokines and recombinant human TG. *IL6* levels were assessed. Means ± standard deviations are shown. *p < 0.05, **p < 0.01, ***p < 0.005.

Because we found that TG is one positive regulator candidate of the IL-6 amplifier (39) and showed that *TG* c.4567C>T (p.1523R>W) allele is increased in MDs with ICRP, we hypothesized that TG is functionally involved in ICRP development via activation of the IL-6 amplifier. Consistently, we found that *TG* expression was increased during the activation of the IL-6 amplifier (Figures 2A, B), suggesting that *TG* is a target gene of the IL-6 amplifier. We then treated H4 cells, a human neuroglioma cell line, with siRNA of *TG* to investigate cytokine-mediated IL-6 amplifier (39) and found that *IL-6* expression and *TG* expression were reduced after cytokine stimulation (Figures 3A, B). Furthermore, recombinant human TG enhanced IL-6 amplifier activation in H4 cells (Figure 3C). These results suggested that TG is both a positive regulator and a target of the IL-6 amplifier.

### 3.3. *TG* is highly expressed and the IL-6 amplifier activation is enhanced in the colon of MD-ICRP with risk allele

**Figure 4:**
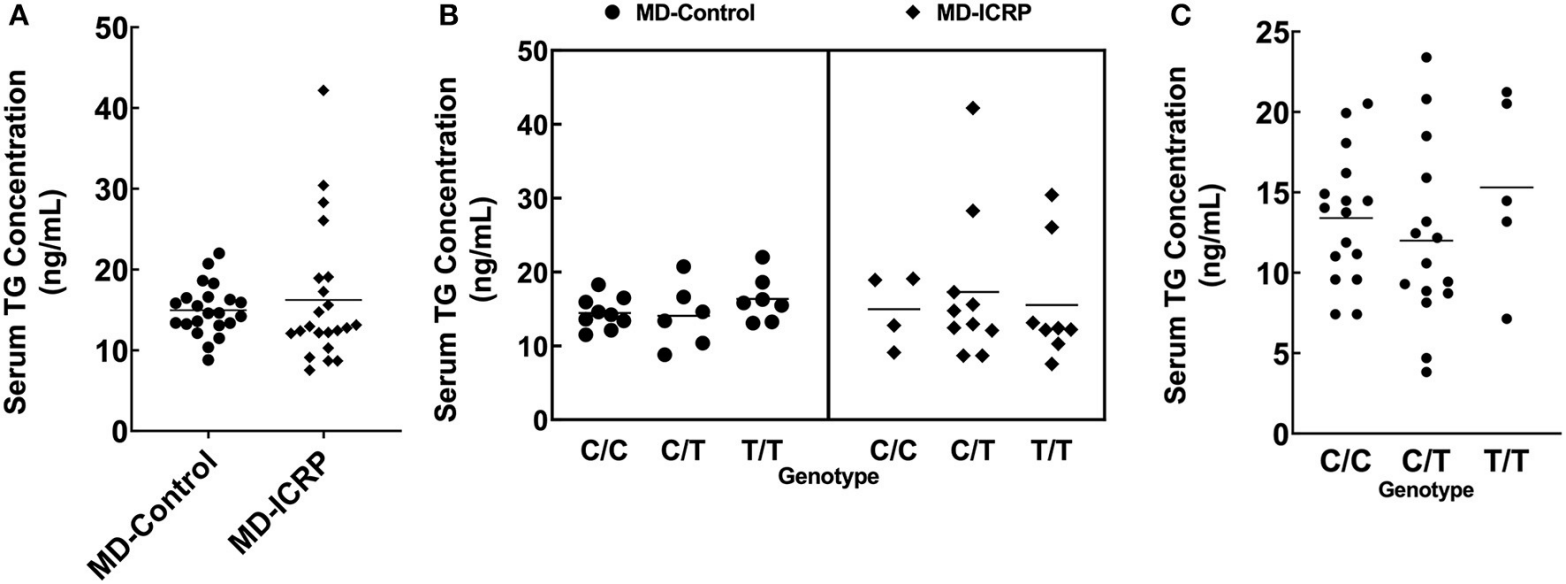
The *TG* SNP does not affect systemic TG levels. **(A)** Serum TG concentration between age-controlled MD-Control and MD-ICRP groups. **(B)** Serum TG concentration between genotypes in MD-Control and MD-ICRP groups. **(C)** Serum TG concentration between genotypes in MDs <1-year-old.

**Figure 5:**
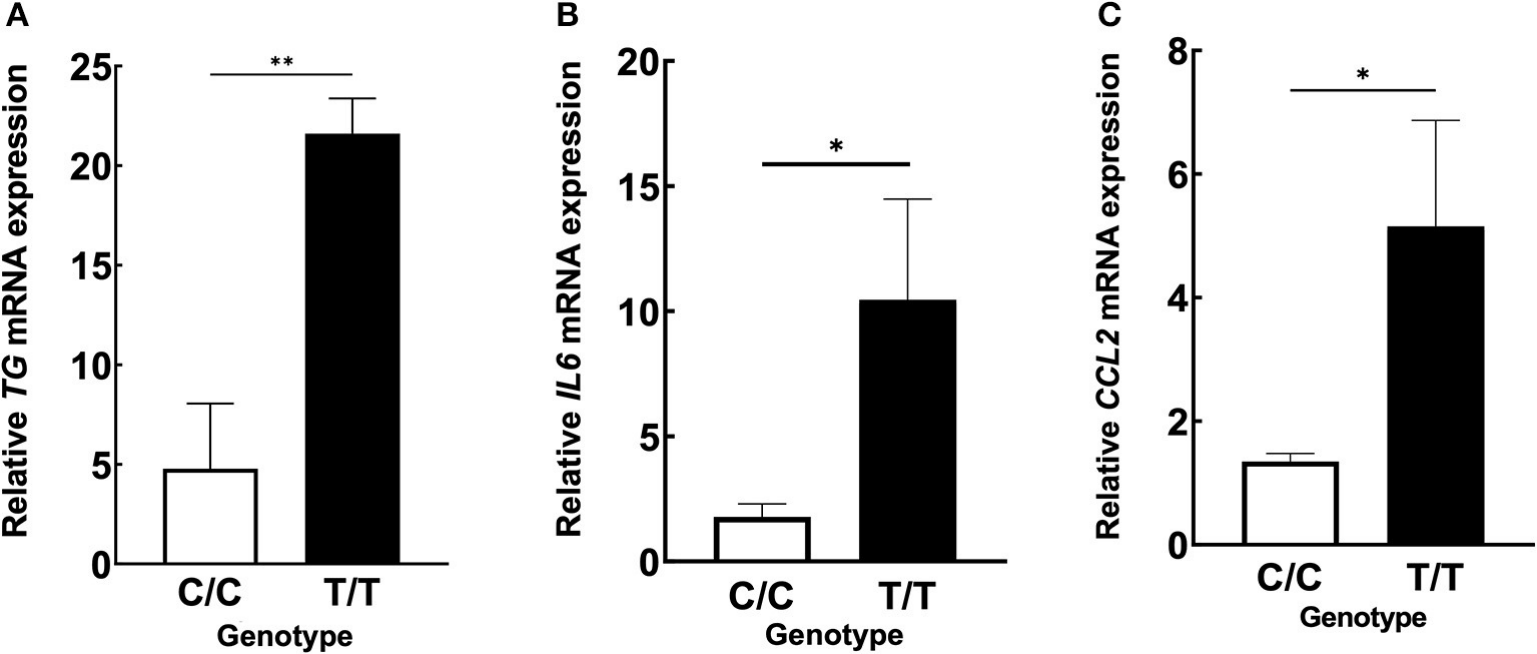
ICRP-MDs with risk alleles have a higher expression of *TG* and NF-κB-related chemokines in non-inflammatory colonic mucosa. **(A)** Relative *TG*, **(B)** *IL6*, and **(C)** *CCL2* mRNA expressions in canine non-inflammatory colonic mucosa adjacent to the ICRP lesion site and diagnosed as normal histopathologically in MDs with wild-type (C/C) and risk (T/T) alleles. Means ± standard error of means is shown. *p < 0.05, **p < 0.005.

We hypothesized that the TG SNP functions to increase *TG* expression either systematically or locally. To test this hypothesis, we investigated the serum concentrations of TG but found they were unchanged in MDs with or without ICRP and with or without the risk T/T SNP (Figures 4A–C). In addition, we found no difference in serum TG concentrations between dogs receiving systemic anti-inflammatory medication or across genotypes (Supplementary Figures 2A–C). We then investigated the expression levels of *TG* in the non-inflammatory colonic mucosa of MD-ICRP. We found that *TG* expression in samples with the risk T/T SNP was significantly higher than those with the non-risk C/C SNP (Figure 5A), suggesting that the TG SNP is an expression quantitative trait locus (eQTL) in the colon of MDs. We next investigated whether *TG* is induced by the IL-6 amplifier in the colon. Because the IL-6 amplifier reflects the activation of NF-κB in non-immune cells, we analyzed the expression of two NF-κB targets, *IL6* and *CCL2*, in the same colon samples. We found that *IL6* and *CCL2* were also enhanced in colon samples with the risk T/T SNP (Figures 5B, C). These results indicate that the risk T/T SNP of *TG* is an eQTL and enhances *TG* expression. They also indicate that TG is involved in the activation of the IL-6 amplifier in the colon of MD-ICRP. Therefore, the risk T/T SNP of *TG* may be critical in the pathogenesis of ICRP through the activation of the IL-6 amplifier.

## 4. Discussion

ICRP has been reported to be breed-associated (1, 44, 45). We recently identified several disease-associated SNPs, such as *PLG, TCOF1, TG, COL9A2*, and *COL4A4*, and found that PLG SNP is associated with IL-6 amplifier activation in the colon (12). In this study, we show that the risk SNP of *TG* is associated with the pathogenesis of ICRP in MDs and that the increased expression of *TG* is associated with the risk allele T/T in the non-inflammatory colonic mucosa, showing that TG possesses a biological role in the activation of NF-κB, which is a critical component of the IL-6 amplifier.

TG is commonly known as the precursor protein to thyroid hormones T3 and T4, which regulate multiple metabolic pathways in the mammalian body (46). Recent advances have shown that TG may function both inside and outside of the thyroid, which is no surprise as *TG* expression was detected in multiple non-thyroidal cells such as human and mouse kidney cells (47). Additionally, *TG* mRNA is expressed in many organs including the testis, suprarenal gland, appendix, lung, and thymus, as well as the hypophysis, lymphocytes, and leukocytes (48–52). Here, we showed that *TG* mRNA is expressed in the colon, especially in MDs with the risk allele T/T of TG SNP. Considering that TG expression with the risk alleles in the colon correlated with the development of ICRPs in MDs, TG-mediated activation of the IL-6 amplifier in the intestine may contribute to the high concurrent occurrence of thyroidal disease and inflammatory bowel disease in human patients (53–56).

Because the local expression of *TG* was higher in the risk allele T/T group compared with the wild-type allele C/C group, we concluded that the function of the SNP is to induce a higher local expression of *TG* in the colon (Figure 5A) but not in the circulation. The localized increase in *TG* mRNA levels was accompanied by localized increases in *IL6* and *CCL2* expression in non-inflammatory colonic mucosa (Figures 5B, C), suggesting that the IL-6 amplifier is activated in the colon of MDs with the risk allele T/T. This result may explain why ICRP only occurs in the colorectal region, although more studies of the expression of TG and other NF-κB targets in other organs, including other regions of the gastrointestinal tract, with or without the risk allele T/T of TG SNP, are needed. Because we found that MDs without the risk allele T/T (C/T or C/C) were affected with ICRP, we hypothesize that one of the reasons is that ICRP in MDs is a polygenic disease and other mechanisms may also be involved in its pathogenesis, although the number of cases for the qPCR analysis in canine colonic mucosa is too small to verify this conclusion. In addition, we are not able to confidently state that there is no blood relationship between the dogs because we do not have the pedigree of each dog used in this study.

In summary, we found a novel SNP variant, *TG* c.4567C>T (p.R1523W), which is involved in the pathogenesis of ICRP in MDs. We also suggested that TG has a functional role in the development of ICRP in MDs through the IL-6 amplifier in colon cells and that the presence of the risk allele T/T correlates with a higher expression of *TG* locally compared to allele C/C. Our results also suggest that the T/T SNP is an eQTL of *TG* mRNA in the colon and the local *TG* expression triggered by this SNP increases the risk of ICRP in MDs via activation of the IL-6 amplifier. Therefore, *TG* c.4567C>T is a potential diagnostic target for ICRP in MDs and *TG*-mediated IL-6 amplifier activation in the colon is a possible therapeutic target for ICRP.

## Data availability statement

Publicly available datasets were analyzed in this study. This data can be found at: https://academic.oup.com/intimm/advance-article/doi/10.1093/intimm/dxad006/7080519.

## Ethics statement

Ethical review and approval was not required for the animal study because samples retrieved from the animals were part of the diagnostic workup for the disease, including blood test, lower gastrointestinal endoscopy, and biopsy and histopathology submission. Written informed consent was obtained from the owners for the participation of their animals in this study.

## Author contributions

MM designed the study and edited the manuscript. J-JJ and YT performed most of the experiments. RH, TY, and NN contributed to the experiments. HO provided the canine samples. KN, NS, and NY advised on the research design. YK confirmed the diagnosis of the canine pathology. YT, MT, and MM wrote the manuscript. MM and MT supervised the study. All authors have read and agreed to the published version of the manuscript.

## Supplementary material

Supplementary Figure 1 **(A)** Sequence chromatograms of *TG* c.4567C>T (p.R1523W) in wild-type, heterozygote, and homozygote variant samples. **(B)** A schematic diagram of the exon of canine *TG* gene and thyroglobulin protein. SP, Signal protein; TGT1, Thyroglobulin Repeat Type 1. **(C)** Alignment of the amino acid sequence in the final thyroglobulin type-1 repeat domain of canine thyroglobulin against selected mammals using the UniProt repository (https://www.uniprot.org/). Asterisks (^*^): complete conservation. Colon (:): strongly similar. Period (.): weakly similar properties. R1523 in dogs is strongly similar to other mammals but is highly conserved among other mammals.

Supplementary Figure 2 The systemic anti-inflammatory drug does not affect systemic TG levels. **(A)** Serum TG concentration between MDs that are treated with or without anti-inflammatory drugs. **(B)** Serum TG concentration between MD-control and MD-ICRP groups that are treated with or without anti-inflammatory drugs. **(C)** Serum TG concentration between genotypes of MDs that are treated with or without anti-inflammatory drugs.

Supplementary Table 1 Details of MDs and other breeds included in this study.

Supplementary Table 2 Case details of MDs recruited for qPCR analysis.

Supplementary Table 3 Case details of MDs recruited for serum TG evaluation.
