# Supplementary figures and images for: An inflammatory bowel disease-associated SNP increases local thyroglobulin expression to develop inflammation in miniature dachshunds
Source: Front Vet Sci. 2023 Jul 14;10:1192888. doi: 10.3389/fvets.2023.1192888 (PMC10375717; doi:10.3389/fvets.2023.1192888)

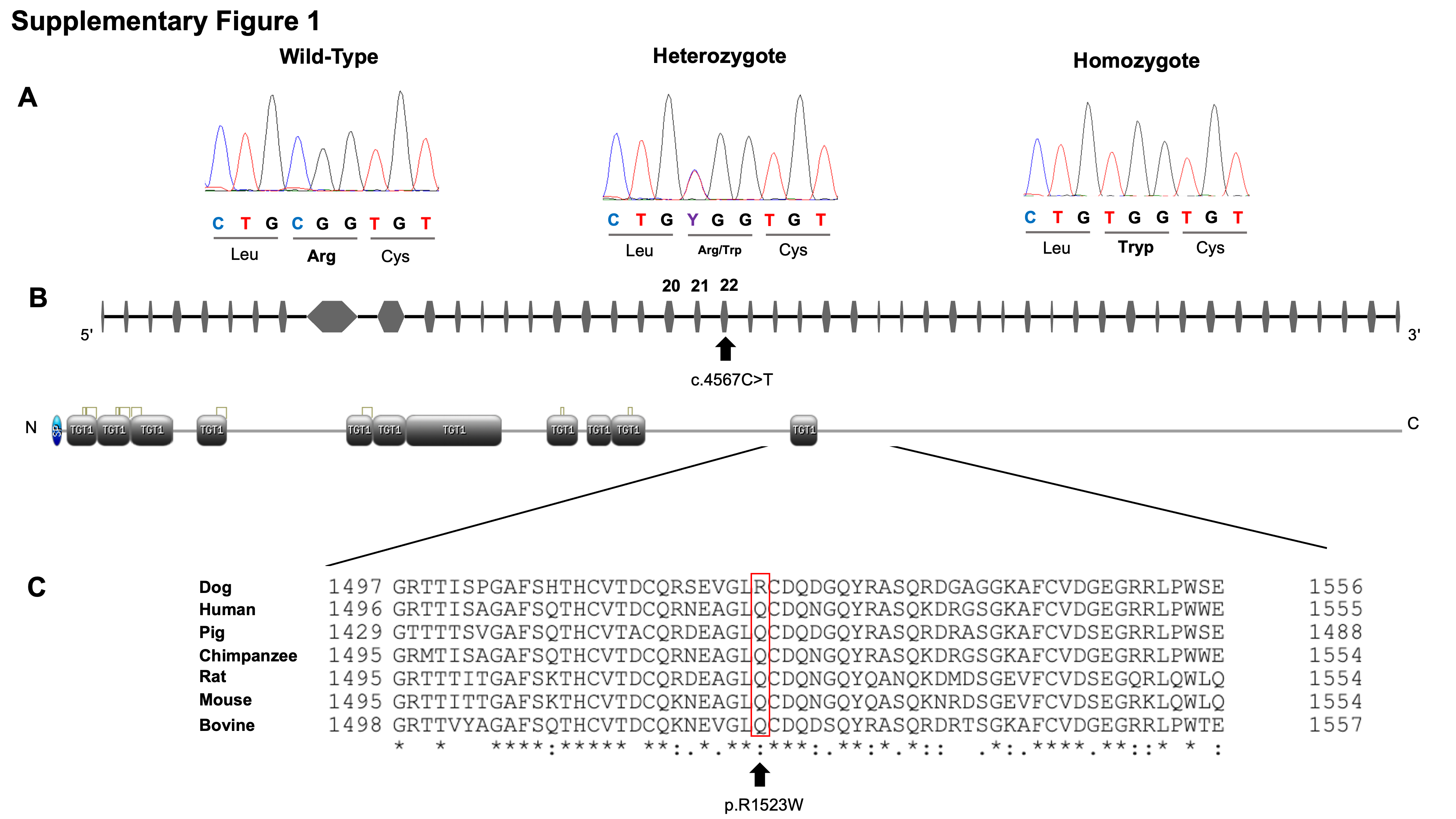

Supplement: Supplementary Figure 1 — (A) Sequence chromatograms of TG c.4567C>T (p.R1523W) in wild-type, heterozygote, and homozygote variant samples. (B) A schematic diagram of the exon of canine TG gene and thyroglobulin protein. SP, Signal protein; TGT1, Thyroglobulin Repeat Type 1. (C) Alignment of the amino acid sequence in the final thyroglobulin type-1 repeat domain of canine thyroglobulin against selected mammals using the UniProt repository (https://www.uniprot.org/). Asterisks (*): complete conservation. Colon (:): strongly similar. Period (.): weakly similar properties. R1523 in dogs is strongly similar to other mammals but is highly conserved among other mammals. [file Image_1.TIFF]

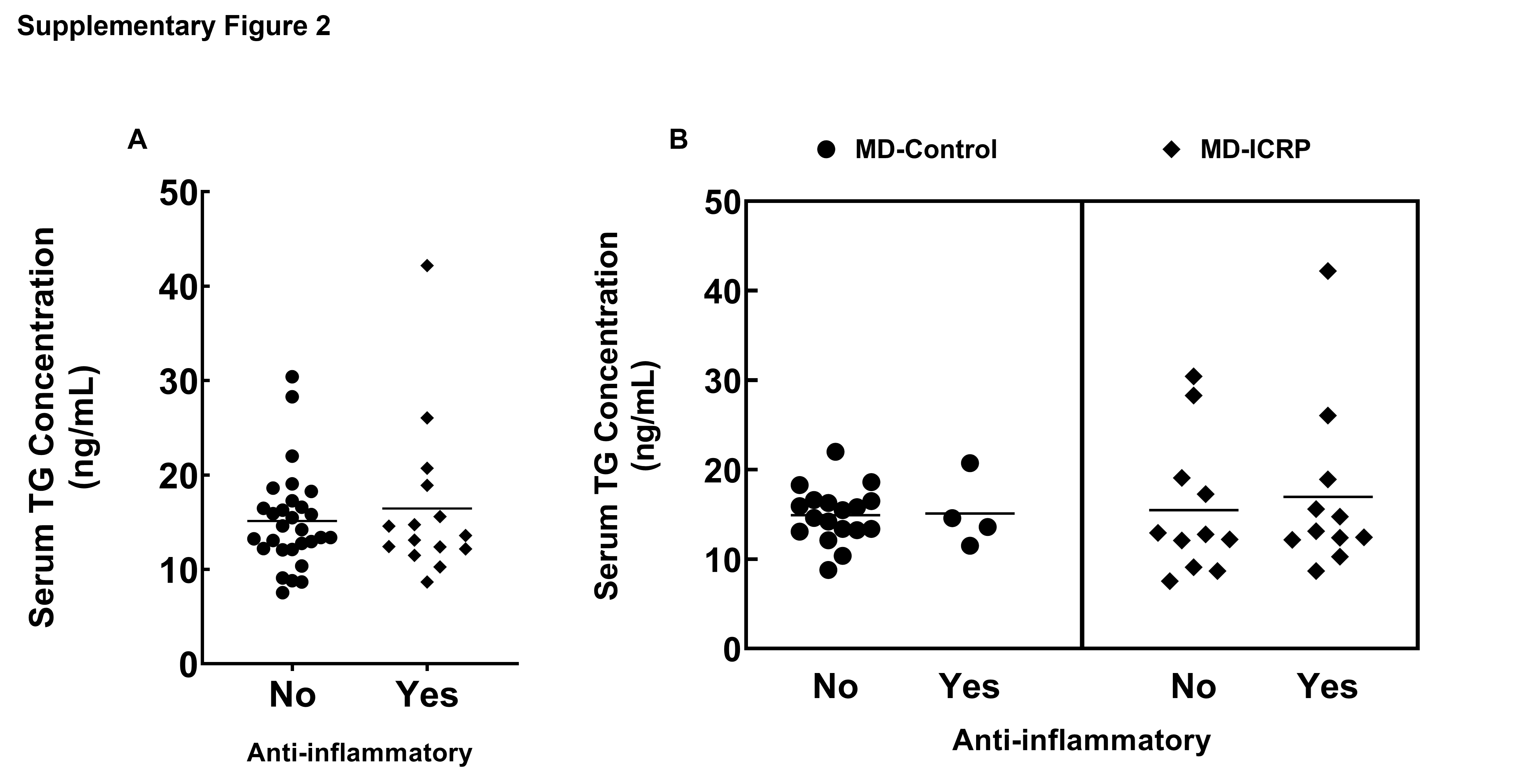

Supplement: Supplementary Figure 2 — The systemic anti-inflammatory drug does not affect systemic TG levels. (A) Serum TG concentration between MDs that are treated with or without anti-inflammatory drugs. (B) Serum TG concentration between MD-control and MD-ICRP groups that are treated with or without anti-inflammatory drugs. (C) Serum TG concentration between genotypes of MDs that are treated with or without anti-inflammatory drugs. [file Image_2.TIF]

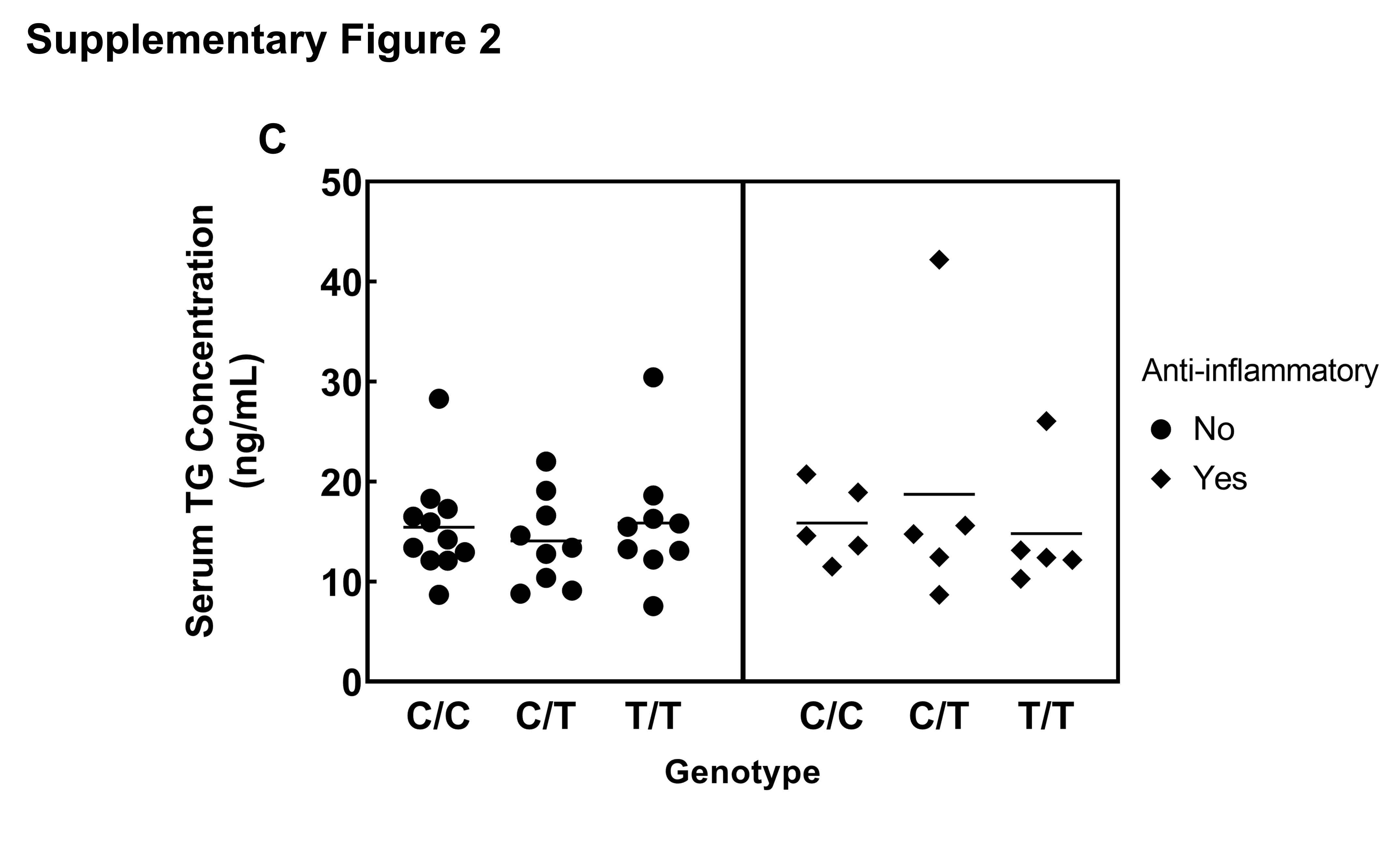

Supplement: Supplementary file 6 [file Image_3.TIF]
